# The effects of immunomodulation by macrophage subsets on osteogenesis in vitro

**DOI:** 10.1186/s13287-016-0276-5

**Published:** 2016-01-22

**Authors:** Florence Loi, Luis A. Córdova, Ruth Zhang, Jukka Pajarinen, Tzu-hua Lin, Stuart B. Goodman, Zhenyu Yao

**Affiliations:** Department of Orthopaedic Surgery, Stanford University School of Medicine, 300 Pasteur Drive, Edwards Building, Room R116, Stanford, CA 94305 USA; Department of Oral and Maxillofacial Surgery, Faculty of Dentistry, University of Chile, Sergio Livingstone Polhammer 943, Independencia, 8380000 Santiago Chile; Department of Bioengineering, Stanford University, 300 Pasteur Drive, Edwards Building, Room R114, Stanford, CA 94305 USA

**Keywords:** Osteoimmunology, Immunomodulation, Macrophage, Preosteoblast, Osteogenesis

## Abstract

**Background:**

Bone formation and remodeling are influenced by the inflammatory state of the local microenvironment. In this regard, macrophages are postulated to play a crucial role in modulating osteogenesis. However, the differential effects of macrophage subsets and their plasticity on bone formation are currently unknown.

**Methods:**

Polarized primary murine macrophages and preosteoblastic MC3T3 cells were co-cultured to investigate the effect of non-activated M0, pro-inflammatory M1, and tissue-regenerative M2 macrophages on the osteogenic ability of MC3T3-E1 cells *in vitro*. Furthermore, to model the physiological transition from inflammation to tissue regeneration, M1-MC3T3 co-cultures were treated with interleukin-4 (IL-4) at different time points to modulate the M1 phenotype towards M2. Macrophage phenotypic markers were assessed by flow cytometry and enzyme-linked immunosorbent assay. A time course study of osteogenic markers at different time points was conducted: alkaline phosphatase (ALP) mRNA levels were evaluated at week 1, ALP activity and osteocalcin and osteopontin mRNA levels at week 2, and matrix mineralization and osteocalcin and osteopontin protein concentrations at week 3. Supernatant collected 72 hours after seeding or IL-4 treatment, whichever was later, was analyzed for oncostatin M, a cytokine released by macrophages that has been recognized to enhance osteogenesis. Unpaired t test or one-way ANOVA with Tukey’s or Dunnett’s post hoc tests were used for statistical comparison of the groups.

**Results:**

Co-culture with any of the macrophage subtypes increased the osteogenic ability of MC3T3 cells as indicated by increases in ALP activity and matrix mineralization. Increased ALP activity, osteocalcin concentration, and matrix mineralization demonstrated that osteogenesis by M1-MC3T3 co-cultures was further enhanced by macrophage phenotype modulation to M2 via IL-4 treatment 72 hours after seeding. Increased oncostatin M protein concentration in untreated M1-MC3T3 co-cultures and M1-MC3T3 co-cultures treated with IL-4 at 72 hours correlated with greater ALP activity and matrix mineralization.

**Conclusions:**

These results suggest that a transient inflammatory phase is crucial for enhanced bone formation. Macrophage plasticity may offer new strategies for modulating the local inflammatory microenvironment with the aim of potentially enhancing bone repair.

**Electronic supplementary material:**

The online version of this article (doi:10.1186/s13287-016-0276-5) contains supplementary material, which is available to authorized users.

## Background

Inflammation, the first stage of healing after tissue injury, can disrupt the delicate balance between bone formation and bone loss in bone remodeling. Macrophages are excellent candidates for immunomodulation of bone regeneration because: they are vital modulators of inflammation [1], their relationship with bone cells enable dynamic crosstalk between the two cell types [2–4], and they are critical for normal bone formation and healing [5–8].

Macrophage populations, which are highly heterogeneous and plastic [9], are broadly described as nonactivated M0 macrophages, which can be classically activated to the proinflammatory M1 phenotype by lipopolysaccharide (LPS) and/or interferon gamma, or alternatively activated to the anti-inflammatory M2 phenotype by interleukin (IL)-4, IL-13, or IL-10 [10, 11]. M1 macrophages initiate the immune response and remove pathogens and tumor cells, while M2 macrophages play central roles in tissue repair and neovascularization [4, 12]. Upon activation, M1 macrophages produce high levels of inducible nitric oxide synthase (iNOS), prostaglandin E2, tumor necrosis factor alpha (TNFα), IL-1β, and IL-6 whereas M2 macrophages express high levels of arginase 1 (Arg1), IL-4, IL-10, IL-1ra, cluster of differentiation 206 (CD206), transforming growth factor beta, and vascular endothelial growth factor [10, 12, 13].

Despite an increasing number of studies confirming the anabolic effect of macrophages on bone formation, a consensus has not been reached on which macrophage phenotype is most beneficial for bone regeneration [14–18]. Whereas the vast majority of in vitro studies in the field of tissue repair highlight the role of macrophage-derived soluble factors on nonosseous single cell cultures (i.e., cardiac tissue repair), few focus on the study of bone tissue repair involving both environmental soluble factors and the direct physical macrophage–osteoblast interactions. Furthermore, to the best of our knowledge, there are no reports investigating the plasticity of macrophage phenotypes with bone therapeutic aims. The aims of the present study were thus to investigate the effects of different macrophage subsets on osteogenic ability of MC3T3 cells, and to assess the temporal role of inflammation on the same outcome via IL-4 modulation of M1 to M2 phenotypes. Here, we show that coculture with any macrophage subset increased bone formation by MC3T3 cells. Additionally, IL-4 treatment of M1-MC3T3 cocultures at 72 hours further enhanced osteogenic ability. We expect that the plasticity of macrophages may provide new opportunities for modulating the local inflammatory microenvironment, potentially enhancing bone repair.

## Methods

### Experimental design

**Fig. 1:**
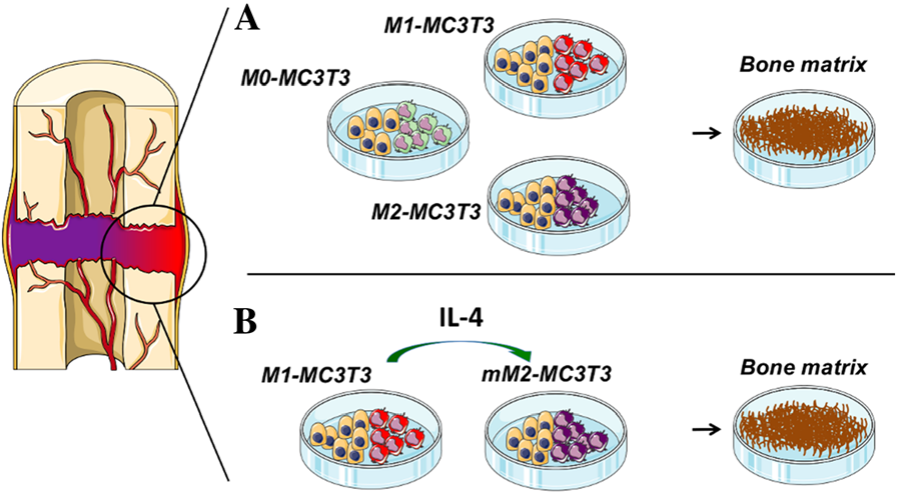
Strategies to assess osteogenic ability by preosteoblasts. M0, M1, and M2 macrophages were directly cocultured with MC3T3 cells in mixed medium **a**. M1-MC3T3 cocultures were treated with IL-4 daily for 72 hours or once at 0, 24, 48, and 72 hours after seeding **b**. *IL* interleukin, *mM2* M2 macrophages modulated from M1 phenotype

The goal of the current study was to evaluate how osteogenesis by MC3T3 preosteoblasts is affected by: coculture with M0, M1, and M2 macrophages (Fig. 1a); and coculture with M1 macrophages modulated to the M2 phenotype at specific time points (Fig. 1b).

### Macrophage polarization

Institutional guidelines for the care and use of laboratory animals were carefully followed in all aspects of this study. Murine bone marrow macrophages were isolated as described by Pajarinen et al*.* [19]. Stanford’s Administrative Panel on Laboratory Animal Care (APLAC) approved this isolation protocol (APLAC 17566). Frozen macrophages were thawed in T-175 culture flasks (8 × 10^6^ cells/flask). Confluent macrophages were polarized to M0, M1, and M2 phenotypes by 24-hour exposure to basal medium (Roswell Park Memorial Institute (RPMI) 1640, 10 % heat-inactivated fetal bovine serum (FBS), 1 % antibiotic/antimycotic (all from Life Technologies, Carlsbad, CA, USA), and 30 % L929 conditioned medium (LCM)), either alone, with 100 ng/ml LPS (Sigma Aldrich, St. Louis, MO, USA), or with 20 ng/ml IL-4 (R&D Systems, Minneapolis, MN, USA), respectively. Macrophage phenotypic markers (iNOS, TNFα for M1; Arg1, IL-1ra, CD206 for M2) for the polarization analyses in this study were selected from the literature [10, 12, 13, 20].

### Macrophage characterization in different media

M0, M1, and M2 macrophages (10^4^ cells/well) were cultured in 24-well plates in MØ medium (RPMI 1640, 10 % FBS, 5 % LCM, 1 % antibiotic/antimycotic, and 1 % GlutaMAX; Life Technologies) for 72 hours. Phenotypic markers were analyzed with flow cytometry and immunofluorescent staining. For immunofluorescent staining, cells were fixed in 2 % paraformaldehyde (PFA)/phosphate-buffered saline (PBS), rinsed in PBS, permeabilized with 1 % saponin/PBS, blocked with 10 % bovine serum albumin/1 % saponin/PBS, incubated at 4 °C overnight with the anti-mouse monoclonal antibodies (mAbs) CD11b-PE (1:80; Biolegend, San Diego, CA, USA), iNOS-Alexa Fluor 488 (1:100; eBioscience, San Diego, CA, USA), and CD206-Alexa Fluor 488 (1:100; Biolegend), and washed with 1 % saponin/PBS and PBS. Cells were mounted with ProLong Gold Antifade Reagent with 4′,6-diamidino-2-phenylindole (DAPI; Life Technologies) and imaged (Axio Observer 3.1; Zeiss, Oberkochen, Germany).

To assess the stability of the macrophage phenotype in the media used in the macrophage/MC3T3 coculture experiments, M0, M1, and M2 macrophages (5 × 10^4^ cells/well) were cultured in 24-well plates in mixed medium (1:1 ratio mix of minimum essential medium (MEMα; Life Technologies) and RPMI 1640 with 10 % FBS, 5 % LCM, 1 % antibiotic/antimycotic, 1 % GlutaMAX, 50 μg/ml l-ascorbic acid (Sigma-Aldrich), 10 mM β-glycerophosphate (Sigma-Aldrich), and 10 nM dexamethasone (Sigma-Aldrich)). Cells were lysed 24 and 72 hours after seeding for quantitative real-time PCR (qRT-PCR) analysis for M1 (iNOS and TNFα) and M2 (CD206 and Arg1) markers. Cell viability 10 days after seeding was determined with the Life Technologies Quant-iT PicoGreen dsDNA Assay Kit.

### RNA isolation and qRT-PCR

RNA was extracted using Qiagen’s RNeasy Mini Kit (Valencia, CA, USA) and reverse transcribed with a High-Capacity cDNA Reverse Transcription Kit (Applied Biosystems, Foster City, CA, USA). qRT-PCR was performed using TaqMan Gene Expression Master Mix and 18s, iNOS, TNFα, CD206, Arg1, osteocalcin (OC), osteopontin (OPN), and alkaline phosphatase (ALP) probes on an ABI 7900HT Sequencing Detection System (all from Applied Biosystems). The GenBank accession numbers of the probe sequences are 18s [NR_003278], iNOS [NM_010927;XM_001001508], TNFα [NM_013693; NM_001278601], CD206 [NM_008625;XM_001003164;XM_001003168], Arg1 [NM_007482], OC [NM_031368], OPN [NM_001204201;NM_001204202;NM_001204203;NM_001204233;NM_009263;XR_106288;XR_106289;XR_107716;XR_107717;XR_107718], and ALP [NM_007431]. 18s rRNA was the internal control. Relative gene expression was quantified with the comparative Ct method.

### Osteogenic differentiation by untreated and IL-4-treated cocultures

Macrophages were polarized prior to plating as described previously. For untreated cocultures, M0, M1, and M2 macrophages (10^4^ cells/well) were seeded simultaneously with MC3T3-E1 subclone 4 cells (10^4^ cells/well; ATCC, Manassas, VA, USA) in 24-well plates in mixed medium (Fig. 1a). For IL-4-treated cocultures, M1-MC3T3 cocultures were set up as described above for six treatment groups: no treatment and IL-4 (20 ng/ml) administered daily for 72 hours or once at 0, 24, 48, or 72 hours after seeding (Fig. 1b). For controls, MC3T3 cells (10^4^ cells/well) were plated in 24-well plates in MC3T3 growth medium (MEMα, 10 % FBS, and 1 % antibiotic/antimycotic) or mixed medium, with or without IL-4 administration at 72 hours. Media were changed twice a week for 3 weeks.

### Flow cytometry

Macrophage phenotypes in the monoculture and cocultures were analyzed by flow cytometry 72 hours after seeding or the last IL-4 treatment, whichever was later. Cells were suspended in 2 % FBS/PBS, preincubated with anti-CD16/32 mAb to prevent nonspecific binding via FcRII/III interactions, and incubated with anti-mouse mAb (CD11b-PE, iNOS-Alexa Fluor 488, and CD206-APC; Biolegend). Appropriate isotypes were used and ethidium monoazide bromide staining excluded dead cells. Analysis was performed on the LSR II Analyzer (BD Immunocytometry Systems, San Diego, CA, USA) in the Stanford Shared FACS Facility, using FlowJo software (TreeStar, Ashland, OR, USA).

### Enzyme-linked immunosorbent assay and ALP activity

TNFα, IL-1ra, oncostatin M (OSM), OPN, and OC secretions were assessed from coculture supernatants by enzyme-linked immunosorbent assay (ELISA) (R&D Systems, Biomedical Technologies, Stoughton, MA, USA). TNFα and IL-1ra levels were determined 72 hours and 1, 2, and 3 weeks after seeding or the last IL-4 treatment, whichever was later. OSM secretions were similarly determined at 72 hours. Late osteogenic markers OC and OPN were analyzed 3 weeks after seeding. Coculture cell lysates were evaluated for ALP activity, an earlier osteogenic marker, using an ALP assay kit (BioAssay Systems, Hayward, CA, USA) 2 weeks after seeding. Manufacturers’ protocols were followed and colorimetric changes were measured with a SpectraMax M2e spectrophotometer (Molecular Devices, Sunnyvale, CA, USA).

### Alizarin Red staining

Three weeks after seeding, cocultures were stained with Alizarin Red (pH 4.1; Sigma-Aldrich) for semiquantitative analysis of bone matrix formation. Plates were scanned with Perfection 1640SU (Epson, Long Beach, CA, USA). All images in their entirety were corrected using Microsoft PowerPoint 2010 (Redmond, WA, USA); brightness and contrast were increased by 20 % and 40 %, respectively. Staining was eluted with 10 % cetylpyridinium chloride (Sigma-Aldrich) and absorbance was measured at 562 nm.

### Statistical analysis

Analyses were performed with GraphPad Prism version 6.04 (San Diego, CA, USA). Unpaired *t* test was used to analyze the difference between groups of two. Analyses for more than two groups were conducted with one-way analysis of variance followed by Tukey’s multiple comparisons test to compare each group (untreated coculture experiment) or Dunnett’s multiple comparisons test to compare with the control group (IL-4-treated coculture experiment). *p* <0.05 was considered statistically significant. Data are the results of at least three independent experiments and are expressed as mean ± standard error of the mean (SEM).

## Results

### Monocultured polarized macrophages increase their proliferation and retain their phenotypes when cultured in mixed medium

First we confirmed the macrophages’ ability to assume expected phenotypes by flow cytometry. After 72 hours of culture in control medium, M0 macrophages were iNOS^low^/CD206^mid^, M1 macrophages were iNOS^high^/CD206^mid^, and M2 macrophages were iNOS^low^/CD206^high^ (Additional file 1: Figure S1). To validate the use of mixed medium to culture polarized macrophages, we assessed their proliferation after 10 days of culture as well as their expression of phenotypic markers after 24 and 72 hours of culture. The proliferation of M0, M1, and M2 macrophages significantly increased in mixed medium (Additional file 2: Figure S2A). At 24 and 72 hours after seeding, no significant differences were found in the relative expressions of phenotypic markers of M1 macrophages (iNOS and TNFα) and M2 macrophages (CD206 and Arg1) attributable to the type of media (Additional file 2: Figure S2B).

### Cocultured polarized macrophages retain their phenotypes and IL-4 effectively modulates cocultured M1 macrophages into M2-like phenotype

To determine whether polarized macrophages also retain their phenotypes in coculture with MC3T3 cells, we analyzed the secretion of macrophage phenotypic markers in culture supernatant up to 3 weeks. At 72 hours and 1 week after seeding, M1-MC3T3 cocultures released higher levels of TNFα, confirming continued M1 polarization (Additional file 3: Figure S3A). Similarly, at all time points, the highest concentration of IL-1ra was released by M1-MC3T3 cocultures (Additional file 3: Figure S3B). Subsequently, both TNFα and IL-1ra secretions decreased in a time-dependent manner. To determine which macrophage subset dominated the inflammatory response at each time point, we calculated the ratio between their phenotypic markers (IL-1ra/TNFα). At 72 hours after seeding, M2-MC3T3 cocultures exhibited the highest IL-1ra/TNFα ratio, demonstrating continued M2 polarization (Additional file 3: Figure S3C). This was confirmed by flow cytometry: at 72 hours after seeding, cocultured M0 macrophages were iNOS^low^/CD206^mid^, M1 macrophages were iNOS^high^/CD206^mid^, and M2 macrophages were iNOS^low^/CD206^high^ (Additional file 3: Figure S3D).

**Fig. 2:**
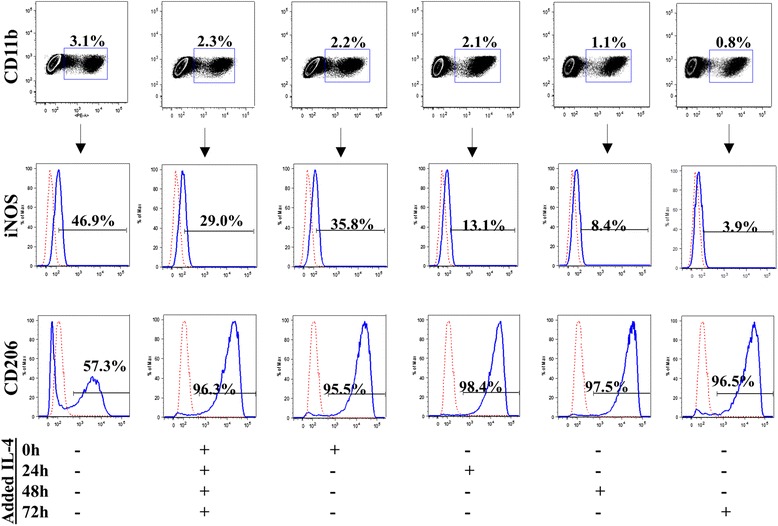
IL-4 treatment of M1-MC3T3 cocultures modulates M1 macrophages to M2 phenotype. Untreated and IL-4-treated M1-MC3T3 cocultures were cultured for 72 hours after seeding or the last IL-4 treatment, whichever was later, and analyzed using flow cytometry. After gating for CD11b^+^ cells, iNOS and CD206 expression was analyzed. *IL* interleukin, *iNOS* inducible nitric oxide synthase

The same analyses were performed to demonstrate the plasticity of cocultured M1 macrophages to modulate their phenotype toward M2 by IL-4 treatment at several time points after the beginning of the coculture (IL-4 administrated daily for 72 hours or once at 0, 24, 48 and 72 hours after seeding). At 72 hours and 1 week after the last dose of IL-4 administration, TNFα secretion, the functional phenotypic marker of M1 macrophages, was significantly decreased in all groups (Additional file 4: Figure S4A). In contrast, at 72 hours after the last IL-4 treatment, the secretion of IL-1ra, the functional phenotypic marker of M2 macrophages, was increased in the groups with IL-4 treatment daily for 72 hours and once at 0 hours and decreased in all other groups (Additional file 4: Figure S4B). However, IL-1ra/TNFα ratios were increased by all IL-4 treatments for 72 hours and 1 week after the last IL-4 treatment, confirming the dominance of M2 phenotype at both time points (Additional file 4: Figure S4C). The IL-4-induced change in macrophage phenotype was again confirmed by flow cytometry: while the percentage of iNOS^+^ cells decreased with IL-4 treatment, the percentage of CD206^+^ cells increased (Fig. 2). The maximum effect of IL-4 treatment was observed in the group with IL-4 administration once at 72 hours after seeding (phenotypic markers: 3.9 % iNOS^+^ and 96.5 % CD206^+^).

### All macrophage subsets enhance osteogenic ability of MC3T3 cells in direct coculture

**Fig. 3:**
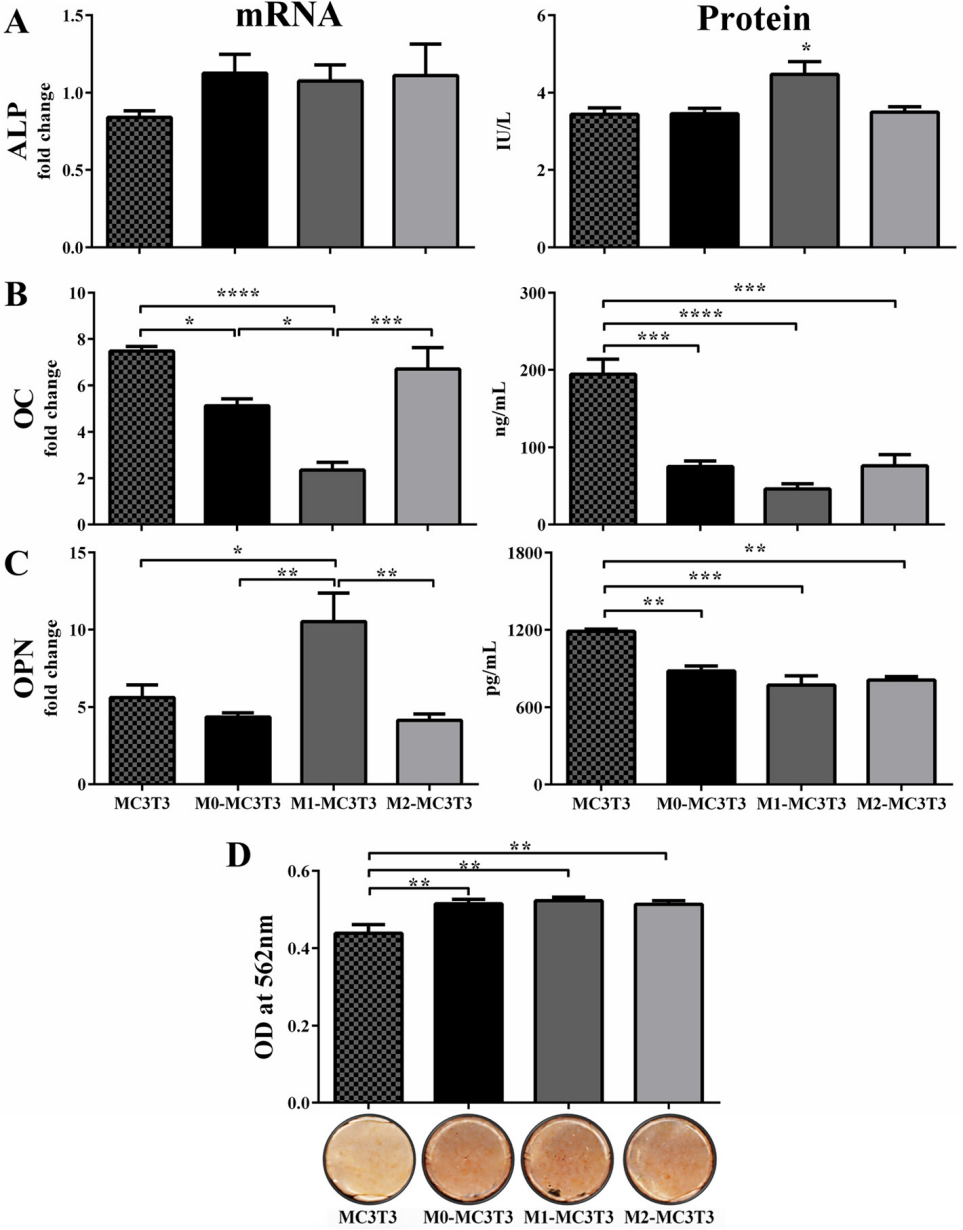
Coculture with macrophages enhances osteogenic ability. MC3T3 cells were cultured alone or with polarized macrophages; analyses were performed 2 or 3 weeks after seeding. Week 2 ALP, OC, and OPN gene expression, relative to housekeeping gene 18s and fold change from MC3T3 monocultures in MC3T3 growth medium, were analyzed by qRT-PCR **a**–**c** (*left panels*). Week 2 ALP activity and week 3 OC and OPN protein secretions were quantified by the *p*-nitrophenyl phosphate method and ELISA, respectively **a**–**c** (*right panels*). Week 3 cultures were stained with Alizarin Red, destained, and quantified by absorbance at 562 nm **d**. *p <0.05, **p <0.01, ***p <0.001, and ****p <0.0001. *ALP* alkaline phosphatase, *OC* osteocalcin, *OD* optical density, *OPN* osteopontin

To analyze the effect of macrophages cocultured with preosteoblasts on the latter’s osteogenic ability, macrophage subsets were directly cocultured in a 1:1 ratio with MC3T3 preosteoblasts. Gene expression and protein levels of ALP, OC, and OPN as well as matrix mineralization were assessed and compared with controls 2 and 3 weeks after seeding. ALP gene expression was enhanced by M0, M1, and M2 macrophages (although not significantly) and ALP enzyme activity was significantly increased in M1-MC3T3 cocultures compared with control (Fig. 3a). On the other hand, we observed that all macrophage phenotypes reduced the OC gene and protein expressions by MC3T3 preosteoblasts except M2 macrophages, which did not significantly decrease OC gene expression (Fig. 3b). Although OPN gene expression was enhanced by M1 macrophages at week 2, OPN protein secretion at week 3 was significantly reduced in all cocultures (Fig. 3c). Finally, by week 3, matrix mineralization was significantly increased in all untreated cocultures compared with controls (Fig. 3d).

### Modulation of macrophage phenotype from M1 to M2 enhance osteogenic ability of MC3T3 cells in cocultures

**Fig. 4:**
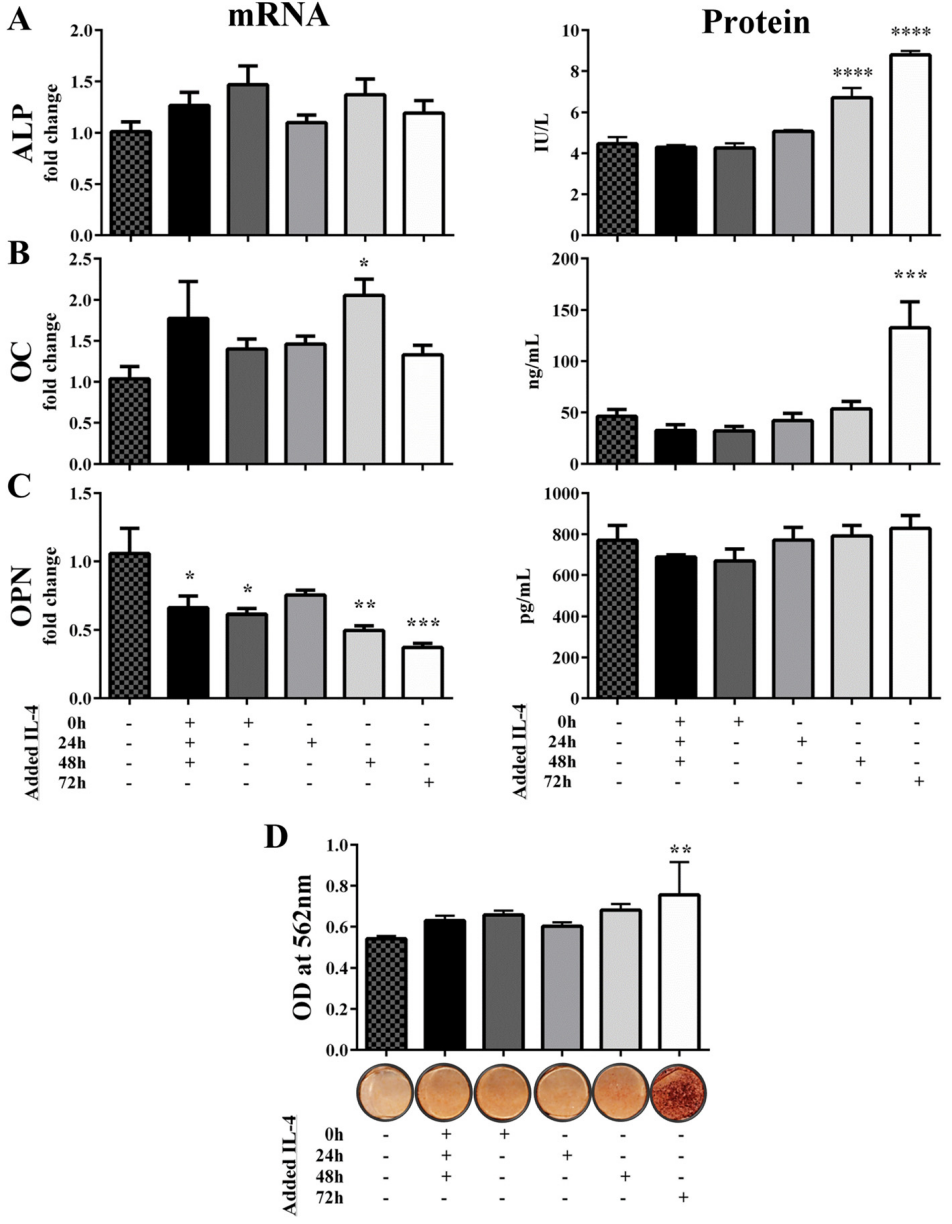
IL-4 treatment of M1-MC3T3 cocultures at 72 hours enhances osteogenic ability. Untreated and IL-4-treated M1-MC3T3 cocultures were analyzed 2 or 3 weeks after seeding. Week 2 ALP, OC, and OPN gene expression, relative to housekeeping gene 18s and fold change from untreated M1-MC3T3 cocultures, were analyzed by qRT-PCR **a–c** (*left panels*). Week 2 ALP activity and week 3 OC and OPN protein secretions were quantified by the *p*-nitrophenyl phosphate method and ELISA, respectively **a–c** (*right panels*). Week 3 cultures were stained with Alizarin Red, destained, and quantified by absorbance at 562 nm **d**. *p <0.05, **p <0.01, ***p <0.001, and ****p <0.0001. *ALP* alkaline phosphatase, *IL* interleukin, *OC* osteocalcin, *OD* optical density, *OPN* osteopontin

Since tissue injury results in transient inflammation followed by a healing phase involving anti-inflammatory mediators, we examined whether modulating the macrophage population from an M1 phenotype to an M2 phenotype would further increase osteogenesis in direct cocultures. To do this, IL-4 was administered to M1-MC3T3 cocultures daily for 72 hours or once at 0, 24, 48, and 72 hours after seeding. Gene expression and protein osteogenic markers were assessed 2 and 3 weeks after seeding. ALP gene expression was enhanced by IL-4 treatments (although not significantly) and ALP protein activity was increased by IL-4 treatment at 48 and 72 hours 2 weeks after seeding (Fig. 4a). Additionally, the week 2 OC gene expression was significantly increased by IL-4 treatment administered 48 hours after seeding and the week 3 OC protein level was very significantly enhanced by IL-4 treatment 72 hours after seeding (Fig. 4b). Week 2 OPN gene expression was decreased in all IL-4 treatment groups (Fig. 4c). However, no change in the week 3 OPN protein level was found in any IL-4 treatment groups. All of this osteogenic activity cumulated in greatest mineralization in M1-MC3T3 cocultures treated with IL-4 72 hours after seeding (Fig. 4d).

To determine whether IL-4 has a direct effect on the osteogenic ability of MC3T3 cells, we treated MC3T3 monocultures with IL-4 72 hours after seeding, which was the administration time point that resulted in the greatest matrix mineralization by M1-MC3T3 cocultures. There were no significant differences in Alizarin Red staining between MC3T3 cells with or without IL-4 treatment in either MC3T3 growth medium or mixed medium (Additional file 5: Figure S5).

**Fig. 5:**
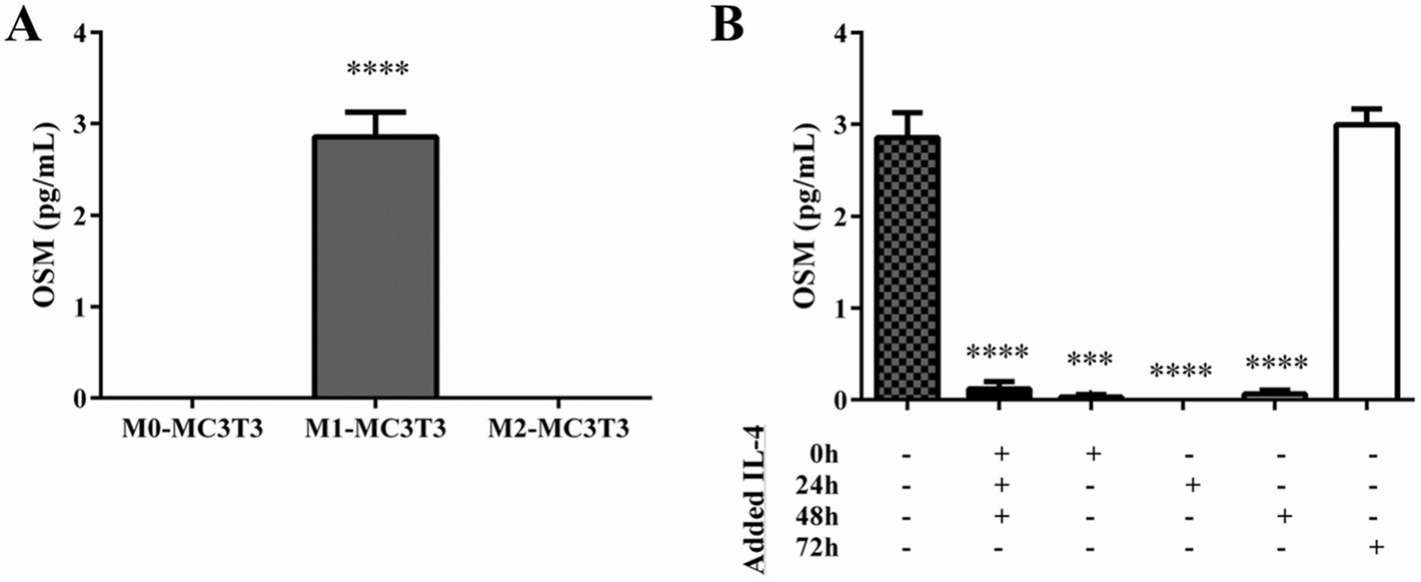
OSM is secreted from M1-MC3T3 cocultures without IL-4 treatment and with IL-4 treatment. OSM protein levels in supernatant collected 72 hours after seeding or treatment from M0-MC3T3, M1-MC3T3, and M2-MC3T3 cocultures **a** and IL-4-treated M1-MC3T3 cocultures **b** were determined by ELISA. ***p <0.001, and ****p <0.0001. *IL* interleukin, *OSM* oncostatin M

We sought to elucidate potential mechanisms for the increased osteoblast differentiation of M1-MC3T3 cocultures when treated with IL-4 72 hours after seeding. Since OSM has been recognized to enhance osteogenesis and macrophages are a potential source of OSM, we assessed OSM secretion in untreated and IL-4-treated cocultures. OSM secretion detected 72 hours after seeding or IL-4 administration was greatly increased in untreated M1-MC3T3 cocultures (Fig. 5a) and in M1-MC3T3 cocultures treated with IL-4 72 hours after seeding (Fig. 5b). IL-4 treatment decreased OSM secretion in M1-MC3T3 cocultures at all other time points.

## Discussion

Bone repair after injuries involves a transient inflammatory response dominated first by M1 macrophages, and second by M2 macrophages leading to the recruitment and activation of osteoprogenitor cells [21]. Greater understanding of these sequential events during the normal pathophysiology of bone repair provides an opportunity to model them experimentally while focusing on innovative treatments for patients with deficiencies in bone repair. This study examined how macrophage subsets affect the osteogenic ability of preosteoblasts via direct coculture of polarized macrophages and preosteoblasts. Furthermore, we assessed the effect of modulating M1 macrophages to assume the M2-like phenotype via IL-4 treatment, which mimics the physiological transition from inflammation to tissue regeneration, on osteogenesis in the same coculture system.

To support both cell types in our coculture system, we proposed the use of culture medium containing promacrophage and proosteogenic factors, which more closely resembles the physiological microenvironment of bone niche during bone healing than typical osteogenic medium containing only proosteogenic factors. Several studies have shown that it is possible to further enhance ascorbic acid, β-glycerophosphate, and dexamethasone-induced MC3T3 cell differentiation and matrix mineralization by adding exogenous growth factors [22, 23]. This, and the fact that mixed medium improved macrophage survival and allowed single and cocultured macrophages to retain their phenotypes, validates the use of mixed medium in our coculture system. Interestingly, while we found no significant difference between the relative expressions of Arg1 by M1 and M2 macrophages cultured in mixed medium versus macrophage medium, the 72-hour Arg1 mRNA expression was strikingly increased in M1 macrophages and markedly decreased in M2 macrophages in both media. Although the literature describes Arg1 induction as a hallmark feature of murine M2 macrophages [24, 25], studies have also reported that LPS-induced M1 macrophages express both iNOS and Arg1 and maximal iNOS expression precedes maximal Arg1 expression [11, 26, 27]. It has been suggested that the late induction of Arg1 functions to downregulate endotoxin-induced nitric oxide production, thus allowing the healing process to commence [27].

Additionally, it is important to note that in our coculture analysis for macrophage phenotypic markers TNFα and IL-1ra, we were unable to separate the cytokines secreted by macrophages and MC3T3 cells because of technical limitations. However, we did find in monocultures that during the first 72 hours MC3T3 cells secreted undetectable TNFα and significantly less IL-1ra than M1 macrophages (although similar to M0 and M2 macrophages; data not shown).

Previous work by our group showed that IL-4, a cytokine which polarizes undifferentiated M0 macrophages to the M2 phenotype [11], can also modulate single cultured M1 macrophages to the M2 phenotype in vitro; in fact, M2 polarization was found to be more efficient if the macrophages were first passed through the M1 polarization state [28]. Additionally, single cultures of M0 and M1 macrophages became M2-like when cultured in supernatant that was subjected to continuous infusion of IL-4 [19]. Here, we demonstrate that IL-4 also modulates cocultured M1 macrophages to M2 macrophages and has no direct effect on the osteogenic ability of MC3T3 cells. Since studies have suggested that some M2 macrophages present at wound healing sites were originally M1 macrophages [29], IL-4 administration allowed us to model this biological scenario in vitro. Furthermore we demonstrated that the modulatory effects of IL-4 were maintained for up to 1 week in cocultures. Therefore, the modulation of macrophage phenotypes from M0 to M2, and now from M1 to M2, by IL-4 administration has been described recently, opening interesting approaches to tissue repair [19, 29, 30].

Since macrophages and preosteoblasts are in close physical contact in the bone niche [31], we sought to determine the impact of this factor on osteogenesis in vitro*.* We demonstrated that the presence of macrophages enhanced bone formation. All macrophage subtypes stimulated greater ALP gene expression and mineralization when cocultured with preosteoblasts. Interestingly, previous research has shown that after removal of polarizing stimuli, macrophages become indeterminate with features of both M1 and M2 phenotypes [32]. It is possible that any differential effect of macrophage subsets on osteogenesis was negated by this phenomenon. However, macrophages retain their phenotypes after at least 72 hours of monoculture in macrophage medium (Additional file 1: Figure S1) and 72 hours of coculture with MC3T3 cells in mixed medium (Additional file 3: Figure S3), as indicated by flow cytometry data. Interestingly, nonactivated M0 macrophages were as capable as M1 and M2 macrophages of promoting matrix mineralization. This may be due to their transient low-level secretion of TNFα, which has been known to promote osteogenesis [14, 33, 34], and the fact that macrophages from the mouse strain C57BL/6 are M1 biased [32]. It is also notable that the increase in osteogenic activity in macrophage–MC3T3 cocultures at week 2 (ALP mRNA and activity, and OPN mRNA) is followed by the reduction of OC and OPN protein secretions at week 3. These discordant results may stem from three sources: the temporal interval between pretranslational and posttranslational processing [35]; the possibility that OPN expression peaked earlier than the time of analysis [36, 37]; and the poorly understood effects of osteogenic factors (β-glycerol phosphate, ascorbic acid, and dexamethasone) on macrophages and MC3T3 cells in a coculture system. Our finding suggests that the macrophages’ effect on osteogenic activity is more essential at week 2; however, further investigation is warranted.

We discovered that a baseline of inflammation before initiation of the healing phase is essential for greater osteogenesis. IL-4 administration allowed us to mimic the biological transition from M1-dominated to M2-dominated macrophage populations at wound healing sites. In our study, osteogenic ability was enhanced only when MC3T3 cells interacted with M1 macrophages for 72 hours before IL-4 modulation, resulting in greater ALP activity, OC secretion, and mineralization detected by Alizarin Red. Thus, in this coculture system, 72 hours of inflammatory signaling is required to promote osteogenesis. Similarly, the addition of low levels of TNFα (≤20 ng/ml), a M1-secreted cytokine, to human adipose tissue-derived mesenchymal stem cells for 72 hours significantly increased osteogenesis in vitro [34]. Our observation is in line with prior work showing cyclooxygenase-2 inhibition results in reduced mineralization by mesenchymal stem cells cultured in conditioned medium from LPS-induced monocytes [4]. Recently, it has been demonstrated that both M1 and M2 macrophages are necessary for enhanced vascularization of tissue engineering scaffolds [30]. The same group is currently developing their scaffolds to promote sequential M1 and M2 macrophage polarization for optimal vascularization [29].

OSM, an inflammatory cytokine released by M1 macrophages, has been implicated in other studies as a key mediator that regulates osteogenic differentiation. Indeed, OSM acts through STAT3 to induce osteoblastic differentiation and mineralization [4]. When cocultured with macrophages, human vascular smooth muscle cells demonstrate calcium deposition attributed to the synergistic upregulation of ALP activity by TNFα and OSM [15, 16]. Here, we show that M1 macrophages do release OSM in coculture. While IL-4 prevents OSM secretion in most treatment groups, however, this is not observed when IL-4 was administered 72 hours after seeding. Our results show that the increase in OSM secretion by these groups was indeed correlated with increased ALP activity and mineralization. Notably, although untreated M1-MC3T3 cocultures secreted elevated levels of OSM compared with M0-MC3T3 and M2-MC3T3 cocultures, no significant difference was found in matrix mineralization. OSM is therefore just one of the several contributing factors for enhanced osteogenesis. The mechanisms in which macrophages promote osteogenesis will be explored in future studies involving transwell systems.

## Conclusions

**Fig. 6:**
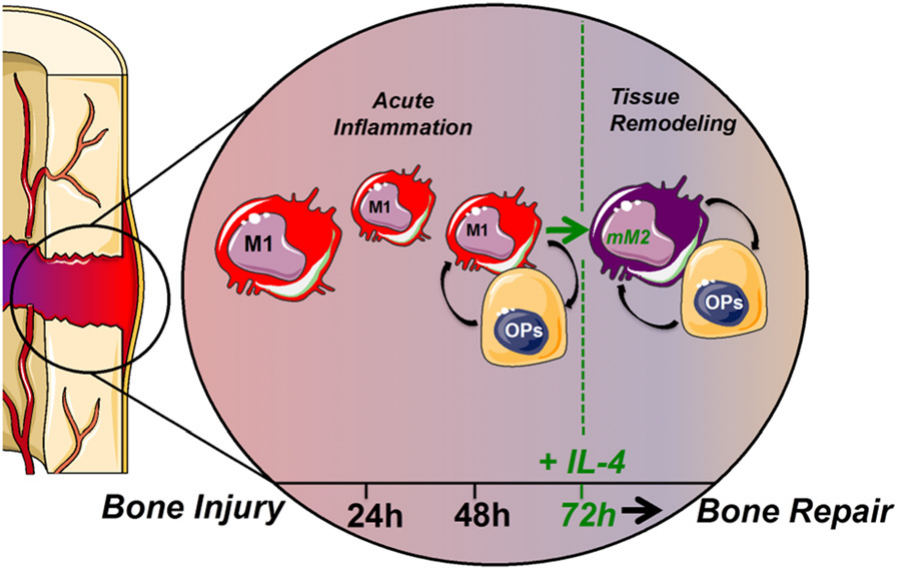
Transient M1 macrophage-driven inflammation is a crucial step for osteogenesis by osteoprogenitors (*OPs*). Delayed (72 hours) phenotypical modulation of M1 macrophages into mM2 macrophages by IL-4 therapy improves osteogenesis by OPs. *mM2* M2 macrophages modulated from M1 phenotype

Taken together, these in vitro results suggest that the macrophages in general promote bone formation. Furthermore, the biological transition from a transient inflammatory to a tissue regenerative microenvironment at bone healing sites is necessary for optimal bone regeneration (Fig. 6). This study provides the biotechnological and scientific foundations for future in vitro and in vivo studies with therapeutic aims of modulating the microenvironment of injury sites for optimal bone healing.

## Additional files

Additional file 1: Figure S1. Showing that macrophages continue to express their respective M1 and M2 markers after 72 hours of culture. Polarized macrophages were labeled with murine CD11b, iNOS, and CD206 mAbs and analyzed by flow cytometry. After gating for CD11b+ cells, iNOS and CD206 expression was analyzed. Immunofluorescent staining of macrophage monocultures was also performed (100X magnification; orange: CD11b, green: iNOS (M1) or CD206 (M2), and blue: DAPI).* iNOS* inducible nitric oxide synthase (TIF 410 kb) Additional file 2: Figure S2. Showing that mixed medium does not adversely affect macrophage viability or phenotype. After 10 days of culture in MØ and mixed media, M0, M1, and M2 macrophage were imaged at 100X magnification and their cell lysates were collected. DNA concentration was determined by PicoGreen test **a**. mRNA was isolated from M0, M1, and M2 macrophages after 24 and 72 hours of culture in MØ and mixed media. Gene expression for iNOS, TNF-α, CD206, and Arg1, relative to housekeeping gene 18s and normalized by M0 macrophages, was analyzed by qRT-PCR **b**. *p <0.05, **p <0.01, ***p <0.001, and ****p <0.0001. *iNOS* inducible nitric oxide synthase (TIF 3354 kb) Additional file 3: Figure S3. Showing that polarized macrophages retain phenotypes in coculture. Supernatant from MC3T3 monocultures and macrophage-MC3T3 co-cultures were collected at 72 hours and weeks 1, 2, and 3 and analyzed for TNF-α **a** and IL-1ra **b** protein levels by ELISA. IL-1ra/TNF-α ratio was calculated **c**. Cells were also labeled with CD11b, iNOS, and CD206 anti-mouse antibodies and analyzed by flow cytometry. After gating for CD11b+ cells, iNOS and CD206 expression was analyzed **d**. *p <0.05, **p <0.01, ***p <0.001, and ****p <0.0001.* IL* interleukin, *iNOS* inducible nitric oxide synthase (TIF 930 kb) Additional file 4: Figure S4. Showing that IL-4 treatment of M1-MC3T3 cocultures modulates M1 macrophages to the M2 phenotype. Supernatant from untreated and IL-4 treated M1-MC3T3 co-cultures and untreated M2-MC3T3 co-cultures were collected at 72 hours and week 1 and analyzed for TNF-α **a** and IL-1ra **b** protein levels by ELISA. IL-1ra/TNF-α ratio was calculated **c**. **p <0.01, ***p <0.001, and ****p <0.0001. *IL* interleukin (TIF 2669 kb) Additional file 5: Figure S5. Showing that IL-4 treatment of MC3T3 cells at 72 hours does not affect their osteogenic ability. Untreated and IL-4 treated MC3T3 monocultures cultured in MC3T3 growth medium or mixed medium were stained with Alizarin Red, destained, and quantified by absorbance at 562 nm. *IL* interleukin, *n.s.* not significant, *OD* optical density (TIF 43 kb)

## Abbreviations

APLAC: Administrative Panel on Laboratory Animal Care
ALP: Alkaline phosphatase
Arg1: Arginase 1
CD206: Cluster of differentiation 206
DAPI: 4′,6-Diamidino-2-phenylindole
ELISA: Enzyme-linked immunosorbent assay
FBS: Fetal bovine serum
iNOS: Inducible nitric oxide synthase
IL: Interleukin
LCM: L929 conditioned medium
LPS: Lipopolysaccharide
mAb: Monoclonal antibody
MEMα: Minimum essential medium alpha
OC: Osteocalcin
OPN: Osteopontin
OSM: Oncostatin M
PBS: Phosphate-buffered saline
PFA: Paraformaldehyde
qRT-PCR: Quantitative real-time PCR
RPMI: Roswell Park Memorial Institute
SEM: Standard error of the mean
TNFα: Tumor necrosis factor alpha
