# Supplementary figures and images for: The effects of immunomodulation by macrophage subsets on osteogenesis in vitro
Source: Stem Cell Res Ther. 2016 Jan 22;7:15. doi: 10.1186/s13287-016-0276-5 (PMC4724110; doi:10.1186/s13287-016-0276-5)

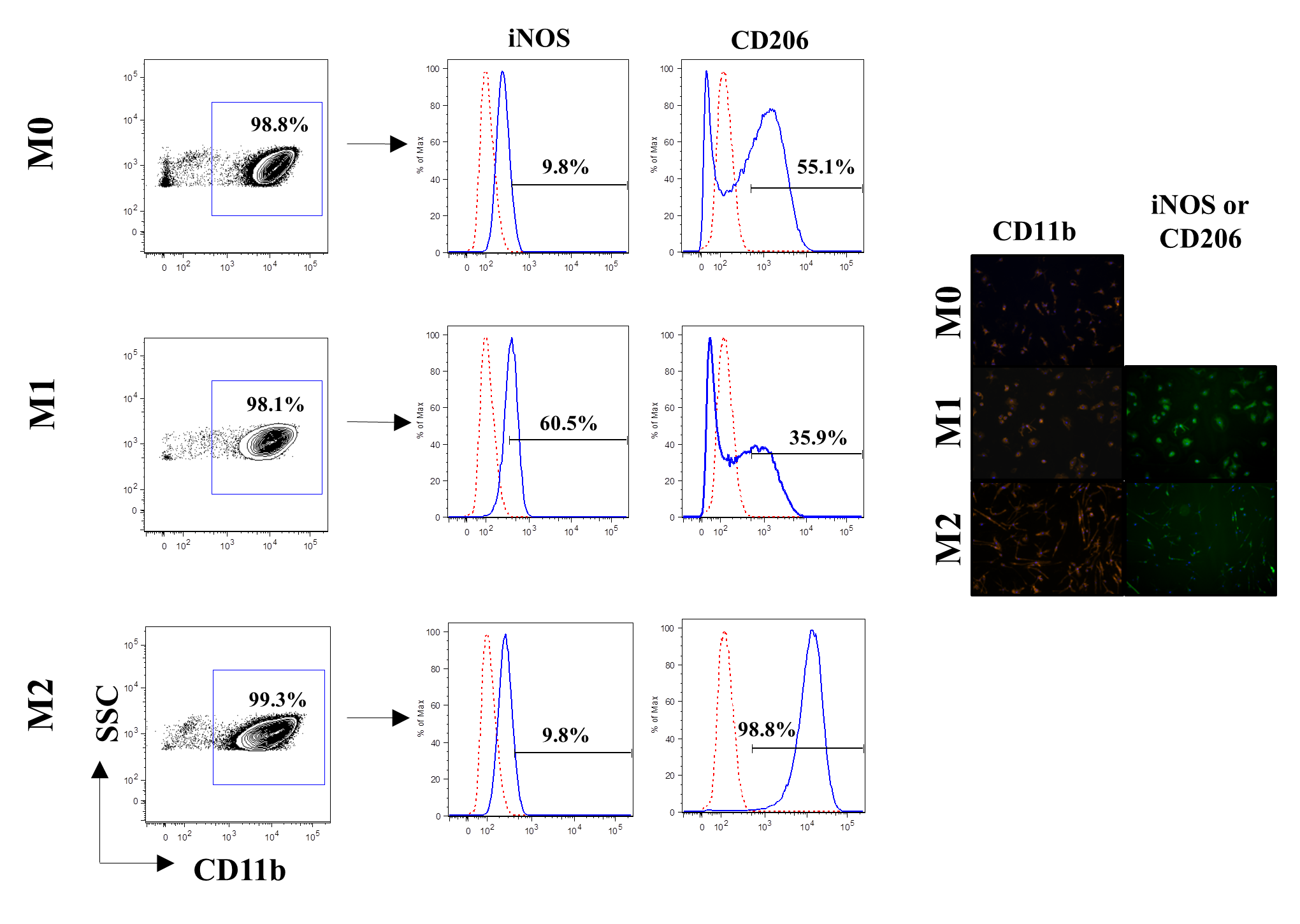

Supplement: Additional file 1: Figure S1. — Showing that macrophages continue to express their respective M1 and M2 markers after 72 hours of culture. Polarized macrophages were labeled with murine CD11b, iNOS, and CD206 mAbs and analyzed by flow cytometry. After gating for CD11b+ cells, iNOS and CD206 expression was analyzed. Immunofluorescent staining of macrophage monocultures was also performed (100X magnification; orange: CD11b, green: iNOS (M1) or CD206 (M2), and blue: DAPI). iNOS inducible nitric oxide synthase (TIF 410 kb) [file 13287_2016_276_MOESM1_ESM.tif]

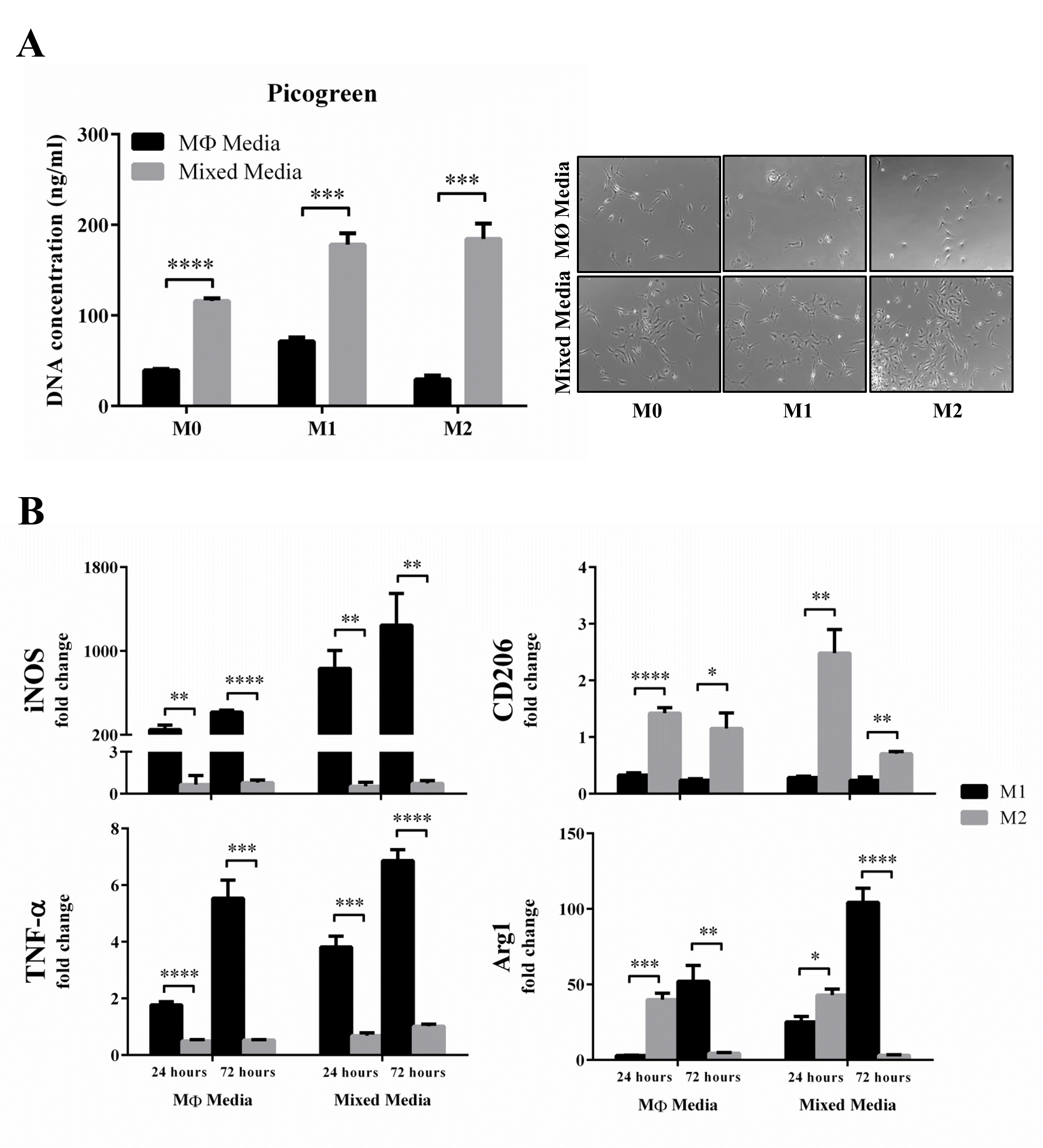

Supplement: Additional file 2: Figure S2. — Showing that mixed medium does not adversely affect macrophage viability or phenotype. After 10 days of culture in MØ and mixed media, M0, M1, and M2 macrophage were imaged at 100X magnification and their cell lysates were collected. DNA concentration was determined by PicoGreen test a. mRNA was isolated from M0, M1, and M2 macrophages after 24 and 72 hours of culture in MØ and mixed media. Gene expression for iNOS, TNF-α, CD206, and Arg1, relative to housekeeping gene 18s and normalized by M0 macrophages, was analyzed by qRT-PCR b. *p <0.05, **p <0.01, ***p <0.001, and ****p <0.0001. iNOS inducible nitric oxide synthase (TIF 3354 kb) [file 13287_2016_276_MOESM2_ESM.tif]

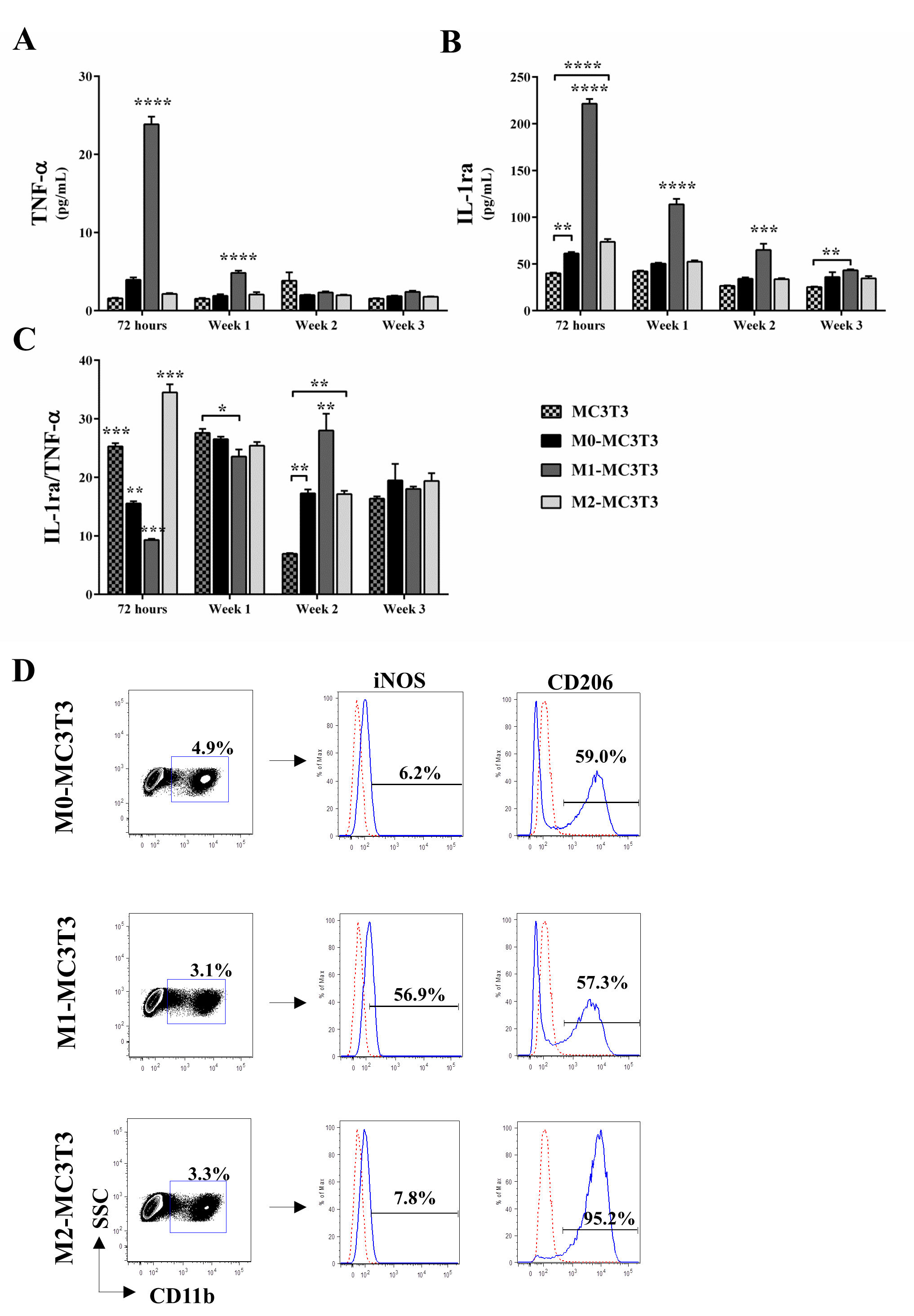

Supplement: Additional file 3: Figure S3. — Showing that polarized macrophages retain phenotypes in coculture. Supernatant from MC3T3 monocultures and macrophage-MC3T3 co-cultures were collected at 72 hours and weeks 1, 2, and 3 and analyzed for TNF-α a and IL-1ra b protein levels by ELISA. IL-1ra/TNF-α ratio was calculated c. Cells were also labeled with CD11b, iNOS, and CD206 anti-mouse antibodies and analyzed by flow cytometry. After gating for CD11b+ cells, iNOS and CD206 expression was analyzed d. *p <0.05, **p <0.01, ***p <0.001, and ****p <0.0001. IL interleukin, iNOS inducible nitric oxide synthase (TIF 930 kb) [file 13287_2016_276_MOESM3_ESM.tif]

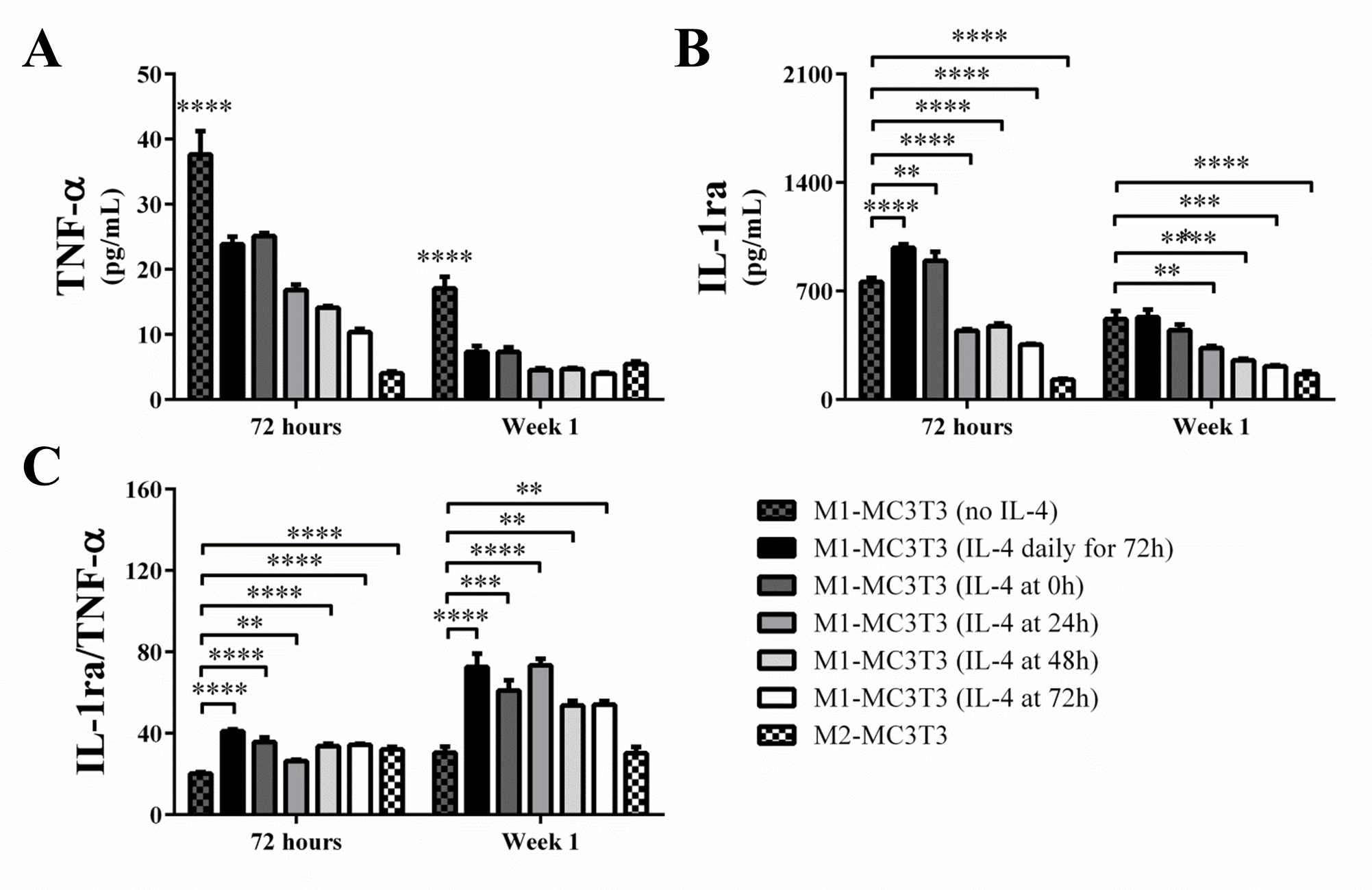

Supplement: Additional file 4: Figure S4. — Showing that IL-4 treatment of M1-MC3T3 cocultures modulates M1 macrophages to the M2 phenotype. Supernatant from untreated and IL-4 treated M1-MC3T3 co-cultures and untreated M2-MC3T3 co-cultures were collected at 72 hours and week 1 and analyzed for TNF-α a and IL-1ra b protein levels by ELISA. IL-1ra/TNF-α ratio was calculated c. **p <0.01, ***p <0.001, and ****p <0.0001. IL interleukin (TIF 2669 kb) [file 13287_2016_276_MOESM4_ESM.tif]
